# Experimental and theoretical studies of Diels–Alder reaction between methyl (*Z*)-2-nitro-3-(4-nitrophenyl)-2-propenoate and cyclopentadiene

**DOI:** 10.1007/s00706-012-0885-3

**Published:** 2013-01-09

**Authors:** Radomir Jasiński, Magdalena Kwiatkowska, Valentin Sharnin, Andrzej Barański

**Affiliations:** 1 Institute of Organic Chemistry and Technology, Cracow University of Technology, Warszawska 24, 31-155 Cracow, Poland; 2 Department of Inorganic Chemistry, University of Chemistry and Technology, F. Engels 7, 153000 Ivanovo, Russia

**Keywords:** Diels–Alder reaction, Nitroalkene, Cyclopentadiene, B3LYP/6–31G(d) calculations

## Abstract

**Abstract:**

The Diels–Alder reaction between methyl (*Z*)-2-nitro-3-(4-nitrophenyl)-2-propenoate and cyclopentadiene yields a mixture of carbodiene Diels–Alder adducts. B3LYP/6–31G(d) simulations indicate that the conversion of addends into methyl (1R*,2S*,3S*,4R*)-2-nitro-3-(4-nitrophenyl)-bicyclo[2.2.1]hept-5-ene-2-carboxylate occurs via two-stage heterodiene Diels–Alder reaction and (in a second step) skeleton rearrangement of the primary cycloadduct, whereas the reaction leading to methyl (1R*,2R*,3R*,4R*)-2-nitro-3-(4-nitrophenyl)-bicyclo[2.2.1]hept-5-ene-2-carboxylate is a single-step process.

**Graphical abstract:**

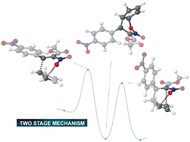

## Introduction

This paper is a follow-up on our studies of the reactivity of conjugated nitroalkenes in Diels–Alder (DA) reactions [1–7]. Previously, we completed detailed kinetic studies and quantum chemical simulations of DA reactions between cyclopentadiene (**1**) and a homogenous series of (*E*)-2-arylnitroethenes [2, 6, 7]. We found that the reactions lead only to carbodiene DA (CDA) reaction products [8–11] and that they occur as one-step, polar (P-DA [12]) processes. Currently, we decided to investigate methyl (*Z*)-2-nitro-3-(4-nitrophenyl)-2-propenoate (**2**), whose π-deficiency is higher than that of (*E*)-2-arylnitroethenes [6], as the dienophile in the reaction with cyclopentadiene. Hence, both carbodiene (CDA, pathways **A** and **B**) and (analogously as suggested by Domingo [13] in the case of DA reaction between nitroethene and dimethylvinylamine) heterodiene [10, 13–16] (HDA, pathways **C–F**) synthesis products could be expected in the cycloadditions of **1** and **2** (Scheme 1).
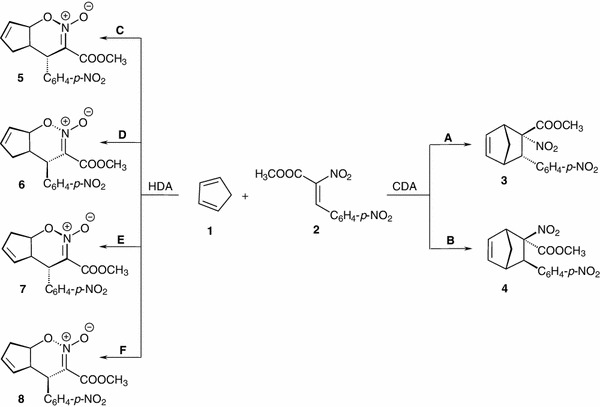


Our intention was to (i) analyse the interactions between addends with respect to the theory of reactivity indices, (ii) determine experimentally which of the theoretically possible reaction pathways really occurs and (iii) suggest a mechanism based on the analysis of critical structures on reaction pathways. We hoped that the compilation of results would lead to a better understanding of the nature of the diene reaction with strongly π-deficient nitroalkenes.

## Results and discussion

To investigate the reaction between cyclopentadiene and methyl (*Z*)-2-nitro-3-(4-nitrophenyl)-2-propenoate more thoroughly before beginning the experimental study, we analysed electronic interactions of the addends using the theory of reactivity indices [17, 18]. Reactivity indices required for this purpose (*μ*, *ω*, *Ν*, *ω*_k_, *N*_k_) were calculated according to equations recommended by Domingo [18, 19].

The comparison of electronic chemical potential values shows that during the cycloaddition **1** + **2** charge transfer should occur from cyclopentadiene (*μ* = −0.1108 a.u.) to nitroalkene **2** (*μ* = −0.1939 a.u.). The global electrophilicity of the nitroalkene (*ω* = 3.38 eV) confirms its strongly electrophilic nature [17]. On the other hand, cyclopentadiene, which is a moderate electrophile (*ω* = 0.83 eV), can be considered as a good nucleophile (*N* = 3.36 eV). The large difference in electrophilicity (∆*ω* = 2.55 eV) of the substrates suggests that—on the basis of Domingo terminology [18]—the reaction **1** + **2** can be considered as a polar DA reaction (P-DA). Analysis of local reactivity indices suggests that the reaction course should be determined by nucleophilic attack from the equivalent 1 or 4 position of cyclopentadiene (*N*_k_ = 0.41 eV) to the *β*-position of nitroalkene (*ω* = 0.18 eV). Such an attack favours reaction channels **A**–**D**.

### Experimental study of the DA reaction of methyl (*Z*)-2-nitro-3-(4-nitrophenyl)-2-propenoate with cyclopentadiene

Before we started investigating the peri- and stereoselectivity of the title reaction, we performed a number of tests to determine its conditions. Therefore, the reactants (**1** + **2**) were heated in sealed ampoules in variable molar ratios, solvent polarity, and ampoule thermostating temperature and time. The composition of the reaction mixtures was monitored using HPLC. Satisfactory reaction progress could be obtained by heating toluene solutions of both reactants at 120–130 °C for 3–4 days in a molar ratio of [**1**]/[**2**] = 4:1. In such conditions, nitroalkene conversion is 75–95 %. After removing the solvent and excess cyclopentadiene under reduced pressure, we performed HPLC analysis of the residue to reveal minute amounts of unreacted nitroalkene **2** (*t*_R_ = 12.7 min), cyclopentadiene dimers (*t*_R_ = 17.3 min) and two other products with different retention times (*t*_R_ = 27.3 min and *t*_R_ = 31.4 min) (in ~1:4.5 ratio). The products were isolated from the reaction mass by semi-preparative HPLC which yielded individual compounds with a purity which enabled all constitutional analyses.

On the basis of the CHN analysis data and the *m*/*z* values of molecular ions (M^+**·**^) read from MS spectra, a formula C_15_H_14_N_2_O_6_ was assigned to both isolated compounds which is exactly the sum of the addends’ molecular weights. The M^+**·**^ ion fragmentation channels are a compilation of fragmentation pathways of norbornene [20, 21], nitrocycloalkane [22, 23] and carboxylic acid ester [22] molecular ions.

**Fig. 1:**
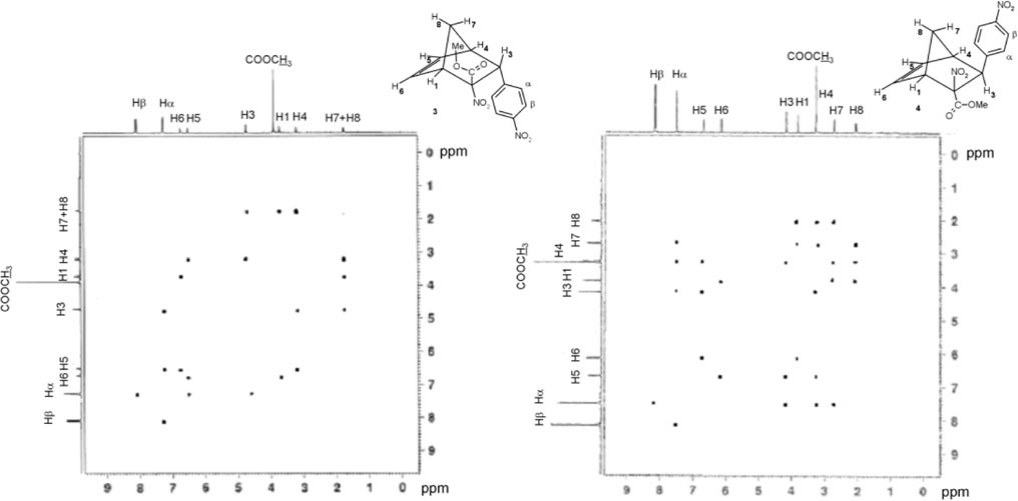
NOESY correlations for methyl (1R*,2S*,3S*,4R*)- and (1R*,2R*,3R*,4R*)-2-nitro-3-(4-nitrophenyl)-bicyclo[2.2.1]hept-5-ene-2-carboxylates **3** and **4** (CDCl_3_, 500 MHz)

Furthermore, their IR spectra reveal absorption bands typical of nitro groups, ester groups and *p*-substituted benzene rings [24]. Bands typical of six-membered cyclic nitrone acid esters are not found [25], which excludes the presence of HDA reaction products in the reaction mass (pathways **C–F**). This is also confirmed by the ^1^H NMR spectra (Fig. 1). The resonance signals in the spectra of the compounds at the weakest field correspond to protons of the *para*-substituted aromatic ring (H_*α*_ and H_*β*_) and protons of the vinyl moiety (H5 and H6) of the molecules. The resonance signals of protons H3 occur at a slightly stronger field. For both isomers, the H1 proton signal occurs at a slightly weaker field than the H4 proton signal. This may be due to the closeness of the NO_2_ and COOCH_3_ groups which cause stronger deshielding than the aromatic ring for the H4 proton. The H7 and H8 proton signals are at the strongest field, typical [26] of bicyclo[2.2.1]heptene rings.

The MS, IR and ^1^H NMR spectra clearly confirm that the reaction tested yields stereoisomeric methyl 2-nitro-3-(4-nitrophenyl)-bicyclo[2.2.1]hept-5-ene-2-carboxylates **3** and **4**, but they do not allow their differentiation. This was solved by recording NOESY spectra of both stereoisomers (Fig. 1).

In the NOESY spectrum of the *t*_R_ = 31.4 min stereoisomer, a correlation signal which proves their spatial closeness is seen at the intersection of projection lines of the H3 and H7 proton chemical shifts onto the spectrum diagonal. However, no correlation signal between the H_*α*_ protons of the aromatic ring and the H7 proton is seen. This suggests that the H3 proton is in the *exo* position, whereas the aromatic ring is in the *endo* position. For the *t*_R_ = 27.3 min stereoisomer, no through-space coupling is seen between protons H3 and H7 in the NOESY spectrum. However, a correlation signal between proton H7 and the H_*α*_ protons of the aromatic ring is seen. This confirms that the H3 proton is in the *endo* position, whereas the aromatic ring is in the *exo* position. Therefore, a structure of methyl (1R*,2S*,3S*,4R*)-2-nitro-3-(4-nitrophenyl)-bicyclo[2.2.1]hept-5-ene-2-carboxylate (**3**) was assigned to the stereoisomer with the higher *t*_R_, and a structure of methyl (1R*,2R*,3R*,4R*)-2-nitro-3-(4-nitrophenyl)-bicyclo[2.2.1]hept-5-ene-2-carboxylate (**4**) to the one with the lower *t*_R_.

Therefore, the experimental studies confirmed that despite the high electrophilicity of nitroalkene **2**, the title reaction occurs periselectively and yields CDA products only. All attempts to find HDA products in the reaction mass were unsuccessful.

### Quantum chemical study of the DA reaction of methyl (*Z*)-2-nitro-3-(4-nitrophenyl)-2-propenoate with cyclopentadiene

The experimental results show that only channels characterized by nucleophilic attack of the equivalent 1 or 4 position of cyclopentadiene to the *β*-position of nitroalkene are feasible. Therefore, we analysed only reaction paths **A**–**D** on the basis of quantum chemical calculations.

All calculations presented in this part of the work were performed on a SGI-Altix-3700 computer in the Cracow Computing Center “CYFRONET”. Hybrid B3LYP functional and 6–31G(d) basis set included within Gaussian 2003 software [27] were applied. The B3LYP/6–31G(d) calculations also nicely illustrate the structure of transition states in cycloadditions between cyclopentadiene and conjugated nitroalkenes. This was also confirmed previously by the compilation of deuterium secondary kinetic isotope effects and results of quantum chemical calculations for cycloaddition between cyclopentadiene and (*E*)-2-phenylnitroethene [7].

For structure optimization of the reactants, pre-reaction intermediates and cycloadducts, the first-order perturbation technique (FOPT) was applied. First-order saddle points were localized using QST2 and Berny procedures. They were verified by diagonalization of the Hessian matrix and by analysis of the internal reaction coordinates (IRC) [28]. Gibbs free energies for all optimized critical structures were computed using data of vibrational analysis. For the calculations of solvents effect, the polarizable continuum model (PCM), in which the cavity is created via a series of overlapping spheres, devised by Tomasi et al. [29, 30] was used. Calculations were performed for *T* = 298 K and *p* = 1 atm. The obtained results are collected in Tables 1 and 2. Consistent with previous conventions [2–4] in this paper the pre-reaction complexes are denoted as **LM** and the transition complexes as **TS**. They are distinguished by appending the letters **A**, **B**, **C** or **D** depending on the reaction pathway.

**Table 1:** Kinetic and thermodynamic parameters of DA reaction of methyl (*Z*)-2-nitro-3-(4-nitrophenyl)-2-propenoate and cyclopentadiene in the gas phase and toluene solution according to B3LYP/6–31G(d) calculations (*T* = 298 K)

Path	Transition	Gas phase			Toluene		
		Δ*H*/kJ mol^−1^	Δ*S*/J mol^−1^ K^−1^	Δ*G*/kJ mol^−1^	Δ*H*/kJ mol^−1^	Δ*S*/J mol^−1^ K^−1^	Δ*G*/kJ mol^−1^
**A**	**1** + **2** → **LM**	−5.4	−114.3	28.5	**–**2.1	−138.1	38.9
	**1** + **2** → **TS-1**_**A**_	77.9	−205.1	139.0	69.5	−201.3	129.3
	**1** + **2** → **5**	−59.4	−213.9	4.2	−61.1	−211.4	1.7
	**1** + **2** → **TS-2**_**A**_	51.5	−230.6	120.1	42.7	−227.3	110.5
	**1** + **2** → **3**	−60.7	−234.0	9.2	−57.8	−231.0	10.9
**B**	**1** + **2** → **LM**	−6.7	−121.8	29.7	−6.3	−148.2	37.7
	**1** + **2** → **TS**_**B**_	87.5	−212.2	150.7	77.9	−207.6	139.8
	**1** + **2** → **4**	−57.8	−231.9	11.3	−54.8	−230.2	13.8
**C**	**1** + **2** → **LM**	−5.4	−114.3	28.5	−2.1	−138.1	38.9
	**1** + **2** → **TS**_**C**_	77.9	−205.1	139.0	69.5	−201.3	129.3
	**1** + **2** → **5**	−59.4	−213.9	4.2	−61.1	−211.4	1.7
**D**	**1** + **2** → **LM**	−4.2	−143.1	38.5	−0.8	−95.8	27.6
	**1** + **2** → **TS**_**D**_	98.4	−202.2	158.6	87.5	−196.3	146.1
	**1** + **2** → **6**	−52.3	**−**218.9	13.0	−90.0	−215.1	−26.0

**Table 2:** Selected physical properties for critical structures of DA reaction between methyl (*Z*)-2-nitro-3-(4-nitrophenyl)-2-propenoate and cyclopentadiene in the gas phase and toluene solution according to B3LYP/6-31G(d) calculations

Solvent	Structure	C4–C5		C6–C1		C2–O7		Δ*l*	*μ*/D	*t*/e	Imaginary frequency/cm^−1^
		*r*/Å	*l* ^a^	*r*/Å	*l*	*r*/Å	*l*				
Gas phase	**1**								6.31		
	**2**								0.44		
	**LM**_**A**_	5.692		5.403		3.741			6.39	0.00	
	**TS-1**_**A**_	3.162		1.993	0.719	2.543	0.259	0.460	8.06	0.33	−336.22
	**5**	3.672		1.555		1.460			6.83	0.37	
	**TS-2**_**A**_	2.587	0.356	1.620		2.456			8.47	0.38	−180.44
	**3**	1.574		1.585					7.58	0.12	
	**LM**_**B**_	3.882		3.825					5.89	0.00	
	**TS**_**B**_	2.728	0.266	1.943	0.761			0.495	9.23	0.29	−339.15
	**4**	1.574		1.569					7.18	0.11	
	**LM**_**C**_	5.692		5.403		3.741			6.39	0.00	
	**TS**_**C**_	3.162		1.993	0.719	2.543	0.259	0.460	8.06	0.33	−336.22
	**5**	3.672		1.555		1.460			6.83	0.37	
	**LM**_**D**_			3.994		3.767			6.45	0.02	
	**TS**_**D**_			1.943	0.757	2.499	0.282	0.475	8.59	0.35	−349.57
	**6**			1.563		1.455			6.75	0.20	
Toluene	**1**								7.18		
	**2**								0.47		
	**LM**_**A**_	3.968		4.097		3.968			7.55	0.01	
	**TS-1**_**A**_	3.197		2.040	0.689	2.602	0.227	0.462	9.45	0.37	−329.20
	**5**	3.673		1.556		1.468			7.87	0.40	
	**TS-2**_**A**_	2.552	0.380	1.620		2.545			10.01	0.43	−164.60
	**3**	1.575		1.585					8.52	0.15	
	**LM**_**B**_	3.892		3.823					6.63	0.00	
	**TS**_**B**_	2.954	0.118	1.949	0.761			0.644	11.33	0.37	−306.53
	**4**	1.569		1.573					8.21	0.15	
	**LM**_**C**_	3.968		4.097		3.968			7.55	0.01	
	**TS**_**C**_	3.197		2.040	0.689	2.602	0.227	0.462	9.45	0.37	−329.20
	**5**	3.673		1.556		1.468			7.87	0.40	
	**LM**_**D**_			4.650		3.846			7.35	0.01	
	**TS**_**D**_			1.956	0.750	2.653	0.196	0.553	10.56	0.41	−328.74
	**6**			1.564		1.471			9.28	0.43	

^a^ $l_{{\text{X - Y}}}  = 1 - \frac{{r_{{\text{X - Y}}}^{{\text{TS}}}  - r_{{\text{X - Y}}}^{\text{P}} }}{{r_{{\text{X - Y}}}^{\text{P}} }}$ where $r^{\text{TS}}_{{{\text{X}} - {\text{Y}}}}$ is the distance between the reaction centres X and Y at the transition structure and $r^{\text{P}}_{{{\text{X}} - {\text{Y}}}}$is the same distance at the corresponding product

#### Reaction profiles

**Fig. 2:**
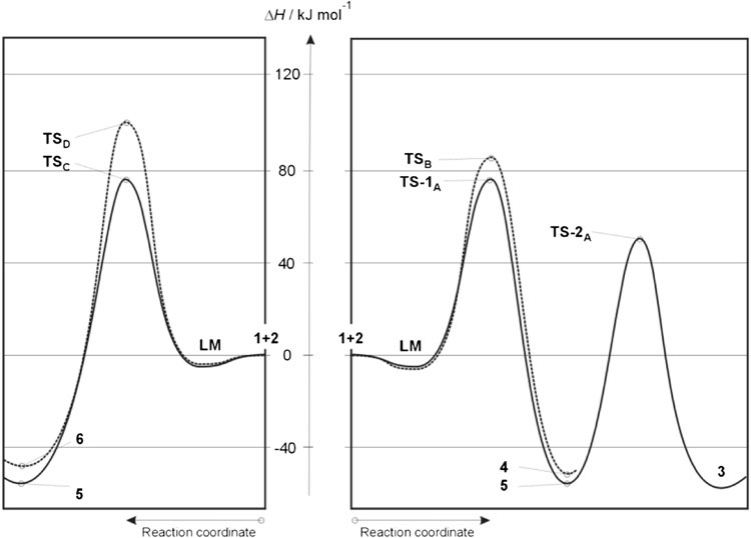
Enthalpy profiles of Diels–Alder reaction between methyl (*Z*)-2-nitro-3-(4-nitrophenyl)-2-propenoate and cyclopentadiene in the gas phase according to B3LYP/6–31G(d) calculations

B3LYP/6–31G(d) calculations prove that the energy profiles of reactions leading finally to CDA products in the gas phase vary. For the pathway leading to **3** (see Table 1, Fig. 2 and Scheme 2) two transition states occur between the minima of reactants **1** + **2** and product **3**. Whereas only one transition state occurs on the pathway leading to **4**. Our attempts to find a pathway leading directly to bicycloheptene **3** were unsuccessful.

**Figure:**
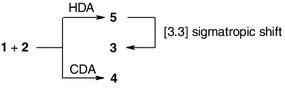

Interactions between addend molecules in the first stage of reaction **1** + **2 **→** 3** lead to the complex **LM**_**A**_. This involves the enthalpy of the reaction system being reduced by 5.4 kJ mol^−1^. The **LM**_**A**_ minimum is exclusively enthalpic in character. As a result of the entropic factor (*Τ*Δ*S*), Δ*G* is greater than zero which excludes the possibility of its existence at *T* = 298 K as a thermodynamically stable intermediate. Further movement of the reaction system along the reaction coordinate leads to a **TS-1**_**A**_ complex; subsequently, the system proceeds to the intermediate, which is methyl 4-aza-2-(4-nitrophenyl)-5-oxybicyclo[3.4.0]nonadiene-3-carboxylate 4-oxide (**5**), previously expected as the final product on reaction pathway **C**. IRC analysis shows that—in Domingo terminology [13]—conversion of addends into **5** may be considered as a one-step, two-stage process, “with concomitant ring closure and without intervention of a zwitterionic intermediate”. When the **TS-1**_**A**_ critical point is achieved, system free enthalpy increases by as much as 139.0 kJ mol^−1^.

The conversion of **5** into **3** occurs as a result of the [3.3]-sigmatropic rearrangement through transition state **TS-2**_**A**_. When achieved, system free enthalpy increases by 120.1 kJ mol^−1^. Therefore, the reaction **1** + **2** → **3** occurs as a two-step process whose rate is limited by the thermodynamic parameters of the critical point **TS-1**_**A**_.

The reaction on pathway **B**, though, occurs as a one-step process. In this case, interactions between the reactants in the first stage of the reaction lead to the complex **LM**_**B**_. This involves the enthalpy of the reaction system being reduced by 6.7 kJ mol^−1^. When the reactant molecules further approach each other, a **TS**_**B**_ complex forms. This involves a system free enthalpy increase of 150.7 kJ mol^−1^. The reaction on pathway **B** is therefore less kinetically favoured than that on pathway **A**. Further movement of the reaction system along the reaction coordinate leads to bicycloheptene **4**.

When toluene (*ε* = 2.38) as a dielectric medium is added to the reaction, the nature of reaction pathways **A** and **B** does not change. Activation barriers only decrease slightly. This, however, does not affect the kinetic pathway preference. Pathway **A** is still more preferred.

HDA reactions (pathways **C**, **D**) in the gas phase occur as one-step processes (Fig. 2). The energy profile of reaction **C** is indeed a copy of the first fragment of the energy profile of reaction **A** (see Fig. 2). The nature of reaction **D** profile is similar, but the quantitative description is different. In particular, reaction occurs through an **LM** complex. Similarly to CDA reactions, **LM**_**D**_ is purely enthalpic. **LM** conversion into product **6** requires an activation barrier related to the formation of a respective **TS** complex to be overcome. The energy expense involved is in this case more than 18.8 kJ mol^−1^ higher than on the preferred pathway **A**. Therefore, pathway **D** may be formally considered kinetically forbidden.

Similarly to CDA reactions, when toluene is added to the medium in HDA reactions, activation barriers are reduced. The reduction, however, is not sufficiently large for pathway **D** to be considered kinetically allowed.

#### Geometries and electronic properties of critical structures

**Fig. 3:**
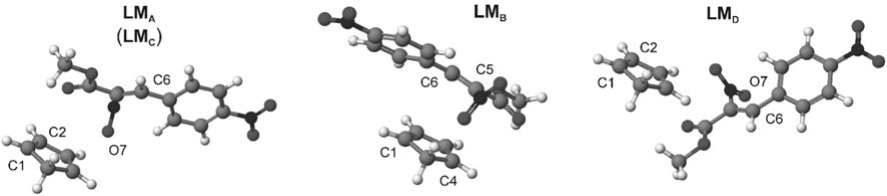
Geometries of pre-reaction complexes for Diels–Alder reaction between methyl (*Z*)-2-nitro-3-(4-nitrophenyl)-2-propenoate and cyclopentadiene in the gas phase according to B3LYP/6–31G(d) calculations

Within the **LM**s, substrates approach each other at a distance of 3.6–5.7 Å. However, their structures have no orientation complex features [31]. The location of reaction centres is different from that observed later in cycloadduct molecules. Furthermore, the **LM**s are not charge transfer complexes [32], because at this reaction stage no charge transfer [33] between substructures occurs (*t* ≈ 0.0 e). When toluene as a dielectric medium is added to the reaction, the geometry of pre-reaction complexes is modified only slightly (Fig. 3)

**Fig. 4:**
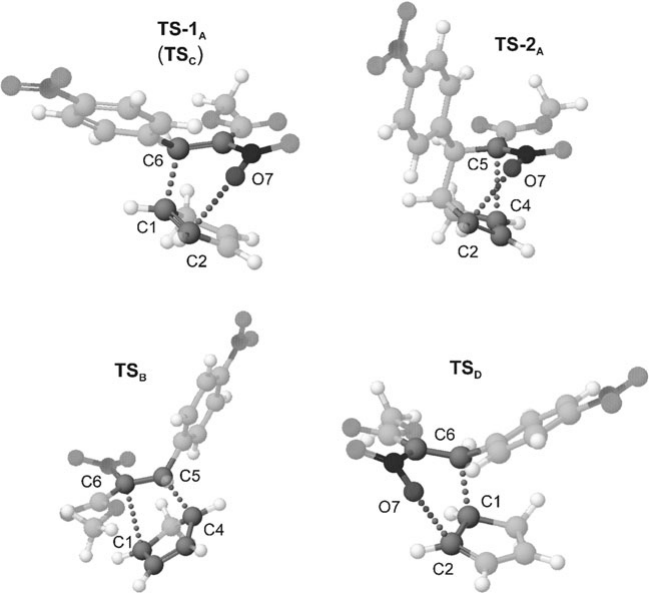
Geometries of transition complexes for Diels–Alder reaction between methyl (*Z*)-2-nitro-3-(4-nitrophenyl)-2-propenoate and cyclopentadiene in the gas phase according to B3LYP/6–31G(d) calculations

The structures of transition complexes in reactions **A**–**D** are much different (Fig. 4). **TS-1**_**A**_ has a biplanar structure, typical of transition complexes in HDA reactions [13, 34]. Both new σ bonds in the complex are formed simultaneously, even though their formation occurs at different rates. The C1–C6 bond, with a length of *r* = 1.993 Å (*l* = 0.719), forms much more rapidly. Simultaneously, the other new σ bond (C2–O7) only starts to be formed. Its length is 2.543 Å (*l* = 0.259). Therefore, the **TS-1**_**A**_ is considerably asymmetric (Δ*l* = 0.460). It is also strongly polar as proved by its dipole moment (*μ* = 8.06 D) and the extent of charge transfer between the substructures (*t* = 0.33 e). Complex **TS**_**D**_ has a similar nature (Δ*l* = 0.475, *t* = 0.35, *μ* = 8.59 D).

The complex **TS**_**B**_ has a biplanar structure as well. Unlike the transition complexes in the HDA reactions discussed earlier, two homoatomic σ bonds form in the complex. Their degree of formation is different: the C1–C6 bond has a length of 1.943 Å (*l* = 0.761), whereas the length of the C4–C5 bond is 2.728 Å (*l* = 0.266). Therefore, the asymmetry of the **TS**_**B**_ complex (Δ*l* = 0.495) is higher than that of the **TS-1**_**A**_ complex. Similarly to the transition complexes in HDA reactions discussed earlier, **TS**_**B**_ also has a strong polar nature (*t* = 0.29 e, *μ* = 9.23 D).

The structure of **TS-2**_**A**_, however, is much different. The complex is highly similar to transition complexes in [3.3]-sigmatropic rearrangements of vinyl allyl ethers [35]. As a result of the circular movement of six π-electrons in the complex, the C2–O7 bond becomes weaker. A σ bond between atoms C4 and C5 forms simultaneously (*r* = 2.587 Å, *l* = 0.356).

When toluene is added to the reaction as a dielectric medium, higher asymmetry of transition complexes on all the pathways tested is found. Their polar nature is also more pronounced, not enough, however, for enforcing a change in the reaction mechanism. Reaction **A** still occurs as a two-step process, whereas reactions **B**–**D** are one-step processes.

**Fig. 5:**
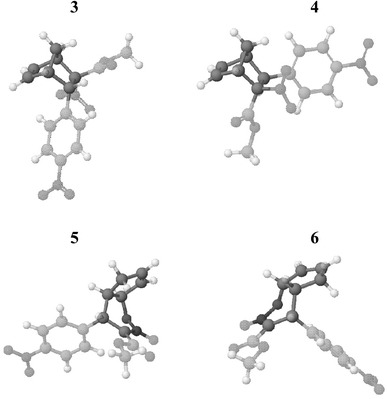
Geometries of products of Diels–Alder reaction between methyl (*Z*)-2-nitro-3-(4-nitrophenyl)-2-propenoate and cyclopentadiene in the gas phase according to B3LYP/6–31G(d) calculations

Within cycloadducts **3**–**6** new σ bonds are already fully formed. The structure of both CDA adducts is typical of norbornenes (see Fig. 5). The distance between H7 and H6 atoms in molecule **3**, relevant for determining the stereoisomerism of the compounds, has a value of 2.45 Å (a correlation signal between protons H6 and H7 is found in the NOESY spectrum), whereas the distance in molecule **4** is as large as 3.76 Å (no correlation signal between protons H6 and H7 is found in the NOESY spectrum). The conformations of HDA adducts, in turn, differ with respect to one another significantly. In particular, bicyclic N-oxide **5** backbone has a “C-like” conformation, whereas N-oxide **6** has a “Z-like” conformation (see Fig. 5).

Thus, the compilation of experimental and quantum chemical results shows that reaction between cyclopentadiene (**1**) and methyl (*Z*)-2-nitro-3-(4-nitrophenyl)-2-propenoate (**2**) proceeds according to the mechanism presented in Scheme 2.

## Conclusion

Despite the high electrophilicity of the nitroalkene, the DA reaction between cyclopentadiene and methyl (*Z*)-2-nitro-3-(4-nitrophenyl)-2-propenoate occurs similarly to those involving less electrophilic *β*-nitrostyrenes. It yields only a mixture of 5-*endo*-nitro-5-*exo*-carbomethoxy-6-*endo*-*p*-nitrophenyl- and 5-*exo*-nitro-5-*endo*-carbomethoxy-6-*exo*-*p*-nitrophenylnorbornenes.

B3LYP/6–31G(d) simulations of theoretically possible reaction pathways indicate that the conversion of addends into methyl (1R*,2S*,3S*,4R*)-2-nitro-3-(4-nitrophenyl)-bicyclo[2.2.1]hept-5-ene-2-carboxylate occurs through 4-aza-2-(4-nitrophenyl)-5-oxybicyclo[3.4.0]nonadiene-3-carboxylate 4-oxide (according to a one-step, two-stage mechanism) which subsequently undergoes [3.3]-sigmatropic rearrangement. However, the reaction leading to methyl (1R*,2R*,3R*,4R*)-2-nitro-3-(4-nitrophenyl)-bicyclo[2.2.1]hept-5-ene-2-carboxylate occurs in one step.

## Experimental

Cyclopentadiene was prepared by pyrolysis of commercially available dicyclopentadiene (Aldrich) at 180–200 °C, according to the standard procedure [36]. Just before use it was distilled under atmospheric pressure, using a 25-cm Vigreux column. Methyl (*Z*)-2-nitro-3-(4-nitrophenyl)-2-propenoate was prepared by reaction of methyl nitroacetate [37] with 4-nitrobenzylideneaniline [38].

For reaction testing, a Knauer apparatus equipped with UV–Vis detector and a Lichrospher 100-5 RP18 column (4 × 250) was applied. Methanol/water mixtures (70:30 v/v) were used as eluent at a flow rate of 0.8 cm^3^ min^−1^. For separation of cycloadducts from the post-reaction mixture, semi-preparative HPLC was applied with a Lichrospher 100-10 RP18 column (16 × 250) and methanol/water (65:35 v/v) as eluent, at a flow rate of 10 cm^3^ min^−1^. Melting points were determined on a Boetius apparatus. IR spectra were recorded on a Bio-Rad 175C spectrophotometer in KBr pellets. UV spectra were taken with a StellarNet EPP-2000C spectrometer in methanol. The mass spectra were obtained using a Finningan 955 apparatus operating at 70 eV ionization energy and 100–130 °C ion source temperature. ^1^H NMR (500 MHz) and ^13^C NMR (125 MHz) spectra were taken on a Bruker AMX 500 spectrometer using CDCl_3_ as the solvent.

### *Synthesis of cycloadducts**** 3**** and**** 4***

A solution of 1 mmol of methyl (*Z*)-2-nitro-3-(4-nitrophenyl)-2-propenoate and 4 mmol of freshly distilled cyclopentadiene in 3 cm^3^ of dry toluene was heated in a sealed ampoule at 125 °C. The reaction was monitored by HPLC at 254 nm wavelength. After 96 h the solvent was evaporated under vacuum and the residue was separated by semi-preparative HPLC. Under these conditions cycloadducts methyl (1R*,2S*,3S*,4R*)-2-nitro-3-(4-nitrophenyl)-bicyclo[2.2.1]hept-5-ene-2-carboxylate (**3**) and methyl (1R*,2R*,3R*,4R*)-2-nitro-3-(4-nitrophenyl)-bicyclo[2.2.1]hept-5-ene-2-carboxylate (**4**) were obtained with a total yield of 89 %.

### Methyl (1R*,2S*,3S*,4R*)-2-nitro-3-(4-nitrophenyl)-bicyclo[2.2.1]hept-5-ene-2-carboxylate (**3**, C_15_H_14_N_2_O_6_)

White crystalline solid; yield 73 %; *t*_R_ = 31.4 min; m.p.: 108–110 °C; IR (KBr): $\bar{\nu }$ = 1,705, 1,537, 1,549, 1,440, 1,347, 740 cm^−1^; ^1^H NMR (CDCl_3_): *δ* = 1.28 (m, 2H, H-7 + H-8), 3.40 (m, 1H, H-4, *J*_4,3_ = 3.1 Hz, *J*_4,5_ = 3.2 Hz), 3.72 (m, 1H, H-1, *J*_6,1_ = 2.8 Hz), 3.89 (s, 3H, OCH_3_), 4.72 (d, 1H, H-3, *J*_4,3_ = 3.1 Hz), 6.51 (m, 1H, H-5, *J*_5,6_ = 5.2 Hz, *J*_4,5_ = 3.2 Hz), 6.83 (m, 1H, H-6, *J*_5,6_ = 5.2 Hz, *J*_6,1_ = 2.8 Hz), 7.16 (d, 2H, H_α_), 8.08 (d, 2H, H_β_) ppm; ^13^C NMR (CDCl_3_): *δ* = 48.0, 49.0, 52.5, 53.9, 54.3, 104.5, 123.4, 129.4, 136.8, 136.9, 145.6, 147.4, 167.3 ppm; MS: *m*/*z* = 318 (M^+^), 272, 271, 256, 212, 66.

### Methyl (1R*,2R*,3R*,4R*)-2-nitro-3-(4-nitrophenyl)-bicyclo[2.2.1]hept-5-ene-2-carboxylate (**4**, C_15_H_14_N_2_O_6_)

White crystalline solid; yield 16 %; *t*_R_ = 27.3 min; m.p.: 65–67 °C; IR (KBr): $\bar{\nu }$ = 1,705, 1,537, 1,542, 1,446, 1,350, 740 cm^−1^; ^1^H NMR (CDCl_3_): *δ* = 2.04 (m, 1H, H-8, *J*_7,8_ = 9.8 Hz), 2.71 (m, 1H, H-7, *J*_7,8_ = 9.8 Hz), 3.25 (m, 1H, H-4, *J*_5,4_ = 2.7 Hz), 3.27 (s, 3H, OCH_3_), 3.82 (m, 1H, H-1, *J*_6,1_ = 3.3 Hz), 4.16 (d, 1H, H-3), 6.15 (m, 1H, H-6, *J*_6,1_ = 3.3 Hz, *J*_5,6_ = 5.7 Hz), 6.70 (m, 1H, H-5, *J*_5,4_ = 2.7 Hz, *J*_5,6_ = 5.7 Hz), 7.52 (d, 2H, H_α_), 8.18 (d, 2H, H_β_) ppm; ^13^C NMR (CDCl_3_): *δ* = 46.0, 48.5, 52.6, 52.8, 53.1, 104.1, 123.4, 129.5, 135.4, 143.0, 145.5, 147.2, 166.4 ppm; MS: *m*/*z* = 318 (M^+^), 272, 271, 256, 212, 66.
